# Montelukast versus inhaled corticosteroids in the management of pediatric mild persistent asthma

**DOI:** 10.1186/2049-6958-7-13

**Published:** 2012-07-05

**Authors:** Alessandra Scaparrotta, Sabrina Di Pillo, Marina Attanasi, Daniele Rapino, Anna Cingolani, Nicola Pietro Consilvio, Marcello Verini, Francesco Chiarelli

**Affiliations:** 1 Allergy and Respiratory Unit, Department of Pediatrics, G. D’Annunzio University of Chieti, Via Dei Vestini 5, Chieti, 66013, Italy; 2 Department of Pediatrics, University of Chieti, G. D’Annunzio University of Chieti, Via Dei Vestini 5, Chieti, 66013, Italy

**Keywords:** Childhood asthma, Inhaled corticosteroids, Leukotriene receptor antagonist, Montelukast

## Abstract

International guidelines recommend the use of inhaled corticosteroids (ICSs) as the preferred therapy, with leukotriene receptor antagonists (LTRAs) as an alternative, for the management of persistent asthma in children. Montelukast (MLK) is the first LTRA approved by the Food and Drug Administration for the use in young asthmatic children.

Therefore, we performed an analysis of studies that compared the efficacy of MLK versus ICSs. We considered eligible for the inclusion randomized, controlled trials on pediatric populations with Jadad score > 3, with at least 4 weeks of treatment with MLK compared with ICS.

Although it is important to recognize that ICSs use is currently the recommended first-line treatment for asthmatic children, MLK can have consistent benefits in controlling asthmatic symptoms and may be an alternative in children unable to use ICSs or suffering from poor growth. On the contrary, low pulmonary function and/or high allergic inflammatory markers require the corticosteroid use.

## Review

Bronchial asthma is a chronic inflammatory disease characterized by airway hyperresponsiveness and respiratory symptoms (breathlessness, wheezing, chest tightness and coughing) [1,2] and the involvement of numerous cell types (eosinophils, T cells, mast cells, basophils and neutrophils) in triggering airway inflammation [3].

Antileukotrienes are a new class of anti-inflammatory drugs, which include Montelukast (MLK), Pranlukast, Zafirlukast and Zileuton, with an important glucocorticoids sparing effects. These drugs interfere either with leukotriene receptors (leukotriene receptor antagonists or LTRAs) or with leukotriene production (5- lipoxygenase inhibitors) [4].

Leukotrienes are important proinflammatory mediators in asthma. These eicosanoids are derived from the metabolism of membrane phospholipids within alveolar macrophages, eosinophils, mast cells and neutrophils, that are involved in the pathophysiology of this disease [5]. Cysteinyl leukotrienes cause bronchoconstriction, mucus secretion, increased vascular permeability and eosinophil migration to the airways, and also promote smooth muscle proliferation. Their synthesis and release appear not to be blocked by corticosteroid therapy [6-12].

MLK is a selective cysteinyl - leukotriene receptor antagonist that reduces asthmatic inflammation and airway resistance and prevents bronchoconstriction [3,13-15]. It is the most studied and used LTRA in pediatric age, being the first approved by the Food and Drug Administration (FDA) for the use in young asthmatic children, at the dosage of 4 mg for children aged from 1 to 5 years and of 5 mg for patients 6 to 14 years of age once daily [16].

It was foreseen that antileukotrienes could be used as the first line agents in the management of mild-moderate persistent asthma. A Cochrane review (last updated in January 2004) summarized the accumulating evidence derived from 13 randomized controlled trials and concluded that low doses of inhaled glucocorticoids were superior to LTRAs [4].

All current international guidelines recommend the use of low-dose (200–400 mcg) of beclomethasone (BDP) or equivalent inhaled corticosteroids (ICSs) as the preferred controller therapy, with LTRAs as an alternative, for the management of persistent asthma in children (5–11 years of age) and adolescents. In patients unresponsive to ICSs alone, the alternative options are the addition of LTRAs or long-acting beta-agonist (LABA), or an increase of ICS dosage [17-20].

Also the pediatric PRACTALL guidelines (PRACTicing ALLergology) [21], in agreement with the GINA (The Global Initiative for Asthma) [18] and the BTS-SIGN (British Thoracic Society Scottish Intercollegiate Guidelines Network) [20] guidelines, indicate ICSs as the first choice for controller therapy of pediatric asthma. The authors of BTS-SIGN guidelines recommend the use of LTRA, if ICS cannot be administered. MLK is the most commonly used in children [22].

ICSs are used as medication for early intervention and long-term management of childhood asthma, because they directly reach the airways and intensively inhibit airways inflammation [23-25]. However, when the amount of drug deposited in the respiratory tract increases with using higher doses, the risks of adverse drug reactions also increase [3,26].

Antileukotrienes have the advantage of being administered orally in a single or twice daily doses and seem to lack the adverse effects on growth, bone mineralisation and on the adrenal axis, associated with long-term systemic glucocorticoid therapy [4]. In general, few patients experienced adverse events during clinical trials with MLK. Headache was the most frequent adverse event; in pediatric patients treated for 8 weeks, diarrhoea, laryngitis, pharyngitis, nausea, otitis, sinusitis and viral infections occurred in more than 2% of MLK recipients and were more prevalent in MLK-treated patients than in placebo recipients [5].

The preference for ICSs is primarily based on evidence from trials comparing mean responses between a treatment group and a control group; however, there is increased appreciation of the considerable interindividual variability in response to ICSs and LTRAs [27-38]. Thus it is important to provide information that can guide the clinician in selecting the most likely medication to achieve a favourable response for particular patients.

Few studies have addressed the factors that determine the marked variability in response to asthma control therapy. It is unknown, for example, whether patients who do not respond well to one medication might respond to another medication [39].

This article reviewed results from randomized, controlled trials of children with mild-moderate asthma in order to assess the usefulness of MLK in the management of persistent asthma compared to the preferred therapy with ICSs.

## Methods

A PubMed search indexed for MEDLINE was undertaken until December 2010, using the keywords “montelukast or leukotriene receptor antagonist and mild persistent pediatric asthma*”* and “montelukast or leukotriene receptor antagonist versus inhaled corticosteroids”, utilizing in the search the limit for age “all child” and/or “randomized controlled trials”.

No time limits were imposed in the search. We have selected 16 randomized, controlled trials performed from 2001 to 2008 on pediatric populations in which LTRAs were compared to ICSs, making a distinction according to MLK efficacy in studies that observed similar results of the two drugs versus others that observed a minor efficacy of MLK compared to ICSs (Tables 1 and 2).

**Table 1:** Studies that demonstrated similar efficacy of MLK compared to ICS

**First author, year [ref]**	**Study duration**	**Patients (Age)**	**Drugs doses**	**Results**
**Maspero, 2001**[40]	6 months	124 pts (6–11 years)	MLK = 5 mg /d IBDP = 300 μg /d	Higher satisfaction for MLK vs IBPD with higher compliance. Similar: oral CS use, safety, FEV_1_ change, asthma-related medical resource utilization, school absenteeism, parental work loss.
**Williams, 2001**[41]	37 weeks	112 pts (6–14 years)	MLK = 5 mg /d IBDP = 300 μg /d	Similar improvement in multiple parameters of asthma control and in daytime symptom scores.
**Stelmach, 2002**[42]	8 weeks	91 pts (12 ± 1.7 years)	TRC = 400 μg /d MLK = 5 mg/d FMT = 24 μg /d	With TRC and MLK: IL-10 level increased, EOS and ECP levels significantly decreased, all clinical parameters improved, with no significant difference in clinical score improvement.
**Karaman, 2004**[43]	14 weeks	63 pts (8–14 years)	MLK = 5 mg /d IBD = 400 μg /d MLK + IBD	MLK improvement: airway obstruction, DSS, β2-a use, nocturnal awakenings, asthma exacerbations, ULKE4 levels.
**Stelmach, 2005**[44]	6 months	51 pts (6–18 years)	IBD = 400 μg /d IBD = 800 μg /d MLK = 5 mg/d	ICS (high dose) and MLK significantly decreased total and specific IgE levels. Clinical score/FEV_1_ significantly improved with medium (p = 0.002) and high dose (p = 0.001) of IBD and MLK (p = 0.002).
**Garcia Garcia, 2005**[45]	12 months	994 pts (6–14 years)	MLK = 5 mg/d FP = 100 μg /d	Significantly greater improvement of RFDs with FP vs MLK, but inferior to the limits (−7%) fixed for judging MLK inferior to FP, so MLK was not inferior to FP in % of asthma RFDs because the adjusted difference was −2.8%.
**Kumar, 2007**[46]	12 weeks	62 pts (5–15 years)	IBD = 400 μg/d MLK = 5 mg/d	The median % predicted FEV_1_ was similar in the two groups (p = 0.44), similar improvement in clinical symptom scores; no significant difference in the need for rescue drugs.
**Stelmach, 2007**[47]	4 weeks	87 pts (6–18 years)	MLK = 5–10 mg /d IBD = 200 μg /d MLK + IBD	Lung function improved significantly in all groups, with no significant difference in improvement.
**Kooi, 2008**[48]	3 months	63 pts (2–6 years)	MLK = 4 mg/day FP = 200 μg/d Placebo	FP had beneficial effect on symptoms (vs placebo, p = 0.021), MLK on EOS vs placebo (p = 0.045). No differences between FP and MLK in lung function parameters, except for FOT.

β_2_-a, β_2_ agonist; DSS, daily symptom scores; ECP, eosinophil cationic protein; EOS, eosinophil blood counts; FEV_1,_ forced expiratory volume in 1 s; FMT, formoterol; FOT, Forced Oscillation Tecnique; FP**,** fluticasone propionate; IBD, inhaled budesonide; IBDP, inhaled beclomethasone; ICS**,** inhaled corticosteroids; MLK, montelukast; pts, patients; RFDs, rescue-free days; TRC, triamcinolone ULKE4, urinary leukotriene E4.

**Table 2:** Studies that demonstrated inferiority of MLK compared to ICS

**First author, year [ref]**	**Study duration**	**Patients (Age)**	**Drugs doses**	**Results**
**Stelmach, 2004**[49]	4 weeks	256 pts (6–18 years)	MLK = 5–10 mg /d TRC = 400 μg /d	With TRC and MLK, FEV_1_ and PC20 significantly increased; mean total symptoms score and EOS significantly decreased. TRC had a stronger effect on PC20 than MLK and in reduction in β_2_-a use, similar improvement in clinical symptoms.
**Ostrom, 2005**[16]	12 weeks	342 pts (6–12 years)	MLK = 5 mg/d FP = 100 μg/d	FP (vs MLK) significantly increased % change from baseline FEV_1_, PEF, % RFDs and reduced night time symptom scores and β_2_-a use.
**Szefler, 2005**[39]	8 weeks	144 pts (6–17 years)	MLK = 5–10 mg/d FP = 200 μg/d	FEV_1_ improvement was 6.8% for FP and 1.9% for MLK (mean difference 4,9%, p = <0,001). ICS therapy is better if low pulmonary function and high levels of allergic inflammation markers.
**Zeiger, 2006**[50]	8 weeks	144 pts (6–17 years)	MLK = 5–10 mg/d FP = 200 μg/d	Significantly greater improvement in ACDs/week with FP than MLK (p = 0.001). Clinical outcomes, pulmonary responses and inflammatory biomarkers improved significantly more with FP than with MLK.
**Sorkness, 2007**[51]	48 weeks	285 pts (6–14 years)	MLK = 5 mg/d FP = 200 μg/d PACT = FP 100 μg + LABA 100 μg/d	Significantly greater improvement with FP vs MLK (p = 0.004). FP group had a longer time to first prednisone burst and to a treatment failure, fewer treatment failure, better FEV_1_, FEV_1_/FVC, PEF, PC20, symptoms score and lower eNO level than MLK group.
**Knuffman, 2009**[52]	48 weeks	191 pts (6–14 years)	MLK = 5 mg /d FP = 200 μg /d PACT combination = FP 100 μg + LABA 100 μg/d	A history of parental asthma best predicted the expected treatment benefit with FP vs MLK in terms of gain in ACDs and time to first exacerbation; elevated baseline eNO predicted response for FP regarding the gain in ACDs; prior ICS use and low PC20 each predicted the expected treatment benefit with FP over MLK regarding time to first exacerbation.
**Szefler, 2007**[53]	52 weeks	395 pts (2–8 years)	MLK = 4–5 mg /d BD = 0,5 mg /d	Both treatments provided acceptable asthma control; however, peak flow and caregiver and Physician Global Assessments favored IBD.

ACD, asthma control days; β_2_-a, β_2_ agonist; eNO, exhaled nitric oxide; EOS, eosinophil blood counts; FEV_1,_ forced expiratory volume in 1 s; FP, fluticasone propionate; IBD**,** inhaled budesonide; ICS**,** inhaled corticosteroids; LABA, long-acting beta-agonist; MLK, montelukast; PEF, peak expiratory flow; pts**,** patients; RFDs, rescue-free days; TRC, triamcinolone.

Inclusion criteria for trials comprised: 1) randomized controlled trials; 2) children aged less than 18 years with a clinical diagnosis of asthma; 3) a minimum of 4 weeks of treatment with MLK compared with ICS; 4) Jadad score [54] > 3; 5) clinical and pulmonary function improvements as outcomes. References of relevant articles were analyzed.

## Results

### Not inferior efficacy of Montelukast compared to Inhaled Corticosteroids

Maspero et al. in a 6-month, open-label extension study compared the efficacy of oral MLK with inhaled BDP (IBDP). A total of 124 out of 266 asthmatic children, 6 to 11 years of age, enrolled in the base study, entered a 6-month open-label extension study (74 boys, 50 girls) and were re-randomized (2:1 ratio) to receive once-daily oral MLK (n = 83) or IBDP 100 mcg three times daily (n = 41). Children and their parents showed a significantly higher overall satisfaction for MLK at 6 months than for IBDP (p = 0.001 and p < 0.05, respectively); they thought that MLK was more convenient (p < 0.001) and less difficult to use (p = 0.005). Oral corticosteroid use was similar in the MLK (13% of patients) and IBDP (17%) treatment groups. There was a higher compliance of patients in the MLK group. The two study groups were similar in safety, change in forced expiratory volume in 1 s (FEV_1_), asthma-related medical resource utilization, school absenteeism and parental work loss. So MLK was considered by the authors as a safe and effective asthma treatment regimen to which children with asthma are more likely to adhere. Conflict of interest was not declared [40].

Another study supporting the similar effectiveness of MLK and ICSs in controlling mild to moderate chronic asthma is that of Williams et al. Treatment with both MLK and ICSs resulted in improvement in multiple parameters of asthma control in 112 children (aged 6–14 years) treated for 37 weeks with IBDP (100 mcg 3 times daily) and MLK (5 mg/daily). Improvements in daytime symptom scores were generally comparable among treatment groups. This study was sponsored by Merck [41].

In three study (2002, 2005, 2007), Stelmach et al. found no statistical significant differences between MLK and ICSs in clinical and functional parameters in children (6–18 years) treated for 8 weeks, 6 months and 4 weeks respectively. In the first one (91 children) the authors found that after treatment with inhaled triamcinolone (Azmacort, Aventis) and MLK the level of IL-10 in blood serum significantly increased, eosinophil blood counts and ECP levels significantly decreased and all clinical parameters improved [42]. In the second one (51 children) they observed that: a high dose of inhaled corticosteroid and MLK significantly decreased levels of total and specific IgE; clinical score and FEV_1_ significantly improved after treatment with medium (p = 0.002) and high dose (p = 0.001) of inhaled budesonide and MLK (p = 0.002). There were no differences between groups in changes of all clinical parameters after treatment [44]. In the third one (87 children), they demonstrated a significant improvement of lung function in all treatment groups. Conflict of interest was not declared [47].

Karaman et al. performed a randomized, 14-week, 2-period, prospective parallel group study in 63 clinically stable outpatients aged 8 to 14 years with a history of mild persistent asthma for at least 1 year. Conflict of interest was not declared. They observed that likewise to inhaled budesonide (IBD), MLK produced improvement in airway obstruction, daily symptom scores, total daily as-needed β-agonist use, nocturnal awakenings, percentage of days and of patients with asthma exacerbations. So, the authors concluded that MLK may be a well-tolerated and effective therapeutic option in 8 to 14-year-old patients with mild persistent asthma [43].

The MOSAIC study (MLK Study of Asthma in Children, sponsored by Merck & Co.) was designed to assess if MLK (5 mg daily, for 12 months) was inferior to inhaled fluticasone propionate (100 mcg 2 times daily, for 12 months) in asthma symptom control [45]. The primary endpoint was the percentage of asthma rescue-free days (RFDs, without rescue medication or asthma-related health care utilization). The two treatments would be considered equivalent if the upper limit of the 95% confidence interval for the difference in the primary endpoint (mean percentages of RFDs) was above −7%. The main conclusion of this study performed on 994 children was that MLK was not inferior to inhaled fluticasone propionate (IFP) in increasing the percentage of rescue-free days, because the adjusted difference was −2.8% (< 1 day per month). The secondary end points (FEV_1_ value, days with β_2_-agonist use, and quality of life) were significantly better in the IFP group, although improved in both groups. In the MLK group there was a major use of systemic corticosteroids (18%) than in the IFP group (11%, p < 0.001). Both treatments were well-tolerated. This study has the criticism that, although the authors calculated the non inferiority interval on the basis of prior studies and before recruitment, the number of asthma rescue-free days clearly showed a difference in favour of IFP (p value not presented) [55,56].

In the study of Kumar et al. MLK demonstrated consistent benefit in controlling symptoms of asthma. The median (95% confidence interval) percentage predicted FEV_1_ was similar for ICSs and MLK after 12 weeks of treatment: FEV_1_ = 76.70 (67.96–90.53) % for IBD; FEV_1_ = 75 (67.40–88.47) % for MLK (p = 0.44). There was similar improvement also in clinical symptom scores and not statistically significant difference between the groups in the need for rescue drugs as well as side effects reported by parents [46].

The study of Kooi et al. [48] was the first, and to date, the only randomized control trial which included preschool children (2–6 years). Conflict of interest was not declared. The study sample was too small, so statistical β-error cannot be excluded. During the study period daily symptom score (DSS) improved in all groups, with statistically significant difference only between IFP and placebo in favour of the first one. This result could be influenced by the higher DSS baseline mean values of the IFP group (3.68) vs MLK group (1.58) (p = 0.02). No differences in DSS final values and in the score of rescue medication use between groups were found. A significant reduction in circulating eosinophils was found in the MLK group only (p = 0.008), which was significantly different from the change found in the placebo group (p = 0.045). So the authors concluded that IFP had a beneficial effect on symptoms and MLK on blood eosinophil level as compared to placebo. Except for a difference in one lung function parameter (frequency dependence, measured by Forced Oscillation Tecnique) after 3 months between IFP and MLK in favour of the IFP group, this study revealed no differences between the two drugs on respiratory function improvement [22].

### Minor efficacy of Montelukast compared to Inhaled Corticosteroids

In contrast with the findings in the other studies, Stelmach et al. in three-arm, randomized no blinding or placebo pragmatic trial compared the effect of a 4-week monotherapy with low-dose of triamcinolone acetonide (400 mcg/day), inhaled nedocromil and MLK on clinical parameters of asthma (score, FEV_1_), bronchial hyperreactivity (PC20/Hystamine), and eosinophil blood count. 256 children, aged 6–18 yr, with mild to moderate asthma, participated in an 8-week study. The study showed the strongest effect of low-dose inhaled steroids on clinical symptoms, lung function, bronchial hyperreactivity and eosinophil blood count when compared to other asthma medications. Conflict of interest was not declared [49].

Ostrom et al. [16] in a controlled study sponsored by a pharmaceutical company (GlaxoSmithKline Inc.) compared the efficacy, safety, health outcomes and cost-effectiveness of three-months treatments of IFP (50 mcg 2 times daily) versus MLK (5 mg daily) in 342 children (6 to 12 years of age) with persistent asthma. Compared with MLK, IFP significantly increased mean percent change from baseline FEV_1_ (p = 0.002), morning PEF (peak expiratory flow) (p = 0.004), evening PEF (p = 0.020), and percent rescue-free days (p = 0.002) at end point, and it significantly reduced night time symptom scores (p < 0.001) and mean total (p = 0.018), and night time (p < 0.001) albuterol use. Parents and physicians satisfaction was higher with IFP [16]. The safety profiles of these drugs were comparable. The costs of the IFP treatments were only one third of those of the MLK treatment [55].

The CLIC study (Characterizing the Response to a Leukotriene Receptor Antagonist and an Inhaled Corticosteroid), sponsored by the National Heart, Lung, and Blood Institute, USA, was an independently funded controlled study that compared the efficacy of ICS and MLK [39,50]. An eight-week cross-over design was used to compare IFP (100 mcg 2 times daily) with MLK (5 mg daily). The main aim of the study was to find predictive factors for a favourable response to either drugs. Improvements in most clinical asthma control measures were seen with both controllers, but all clinical outcomes, pulmonary responses (the mean percentage of improvement in FEV_1_ was 7% for IFP and 2% for MLK, p < 0.001), and inflammatory bio-markers improved significantly more with IFP than with MLK treatment. A favourable response to IFP alone (23% of subjects) was associated with higher levels of eNO (exhaled nitric oxide), total eosinophil counts, higher levels of serum IgE and higher levels of serum eosinophil cationic protein, and lower levels of methacholine PC20 (the provocation concentration of inhaled methacholine that causes a 20% decrease in FEV_1_) and decreased pulmonary function values. A favourable response to MLK alone (5% of subjects) was associated with lower age and shorter disease duration. Finally, greater differential response to IFP over MLK was associated with higher bronchodilator use, bronchodilator response, eNO levels and eosinophil cationic protein levels, lower methacholine PC20 and pulmonary function values [55].

When asthma control days (ACDs) were used as an outcome, higher baseline eNO levels, greater salbutamol use, and more positive aeroallergen skin test responses, in addition to fewer ACDs at baseline, predicted more ACDs variations after IFP treatment [50,55]. For MLK no predictor, except fewer ACDs at baseline, was associated with more ACDs during treatment. Higher eNO levels at baseline was the only baseline characteristic discriminating the ACD response to treatments and was positively associated with greater ACD responses to IFP than to MLK. No difference in adherence to medications was found, but dropouts were more common in the MLK group. The authors concluded that asthma therapy may soon move from the current approach, based on mean responses in populations, to one in which the treatment that is the most likely to rapidly produce a favourable response is identified in each individual patient on the basis of their phenotypic and, possibly genotypic, characteristics [55].

The PACT study (Pediatric Asthma Controller Trial), sponsored by the National Heart, Lung, and Blood Institute, was another independently funded controlled study, in which a total of 285 children (ages 6–14 years) with mild to moderate persistent asthma were randomized to 1 of 3 double-blind 48-week treatments: IFP 100 mcg twice daily, IFP 100 mcg/salmeterol 50 mcg in the morning and salmeterol 50 mcg in the evening (PACT combination), and MLK 5 mg in the evening. The outcomes included asthma control days (primary outcome), exacerbations, humanistic measurements, and pulmonary function measurements. IFP monotherapy and PACT combination were comparable in many patient-measured outcomes, including percent of asthma control days, but IFP monotherapy was superior for clinic-measured FEV_1_/forced vital capacity (p = 0.015), maximum bronchodilator response (p = 0.009), eNO (p < 0.001), and methacholine PC20 (p < 0.001). IFP monotherapy was superior to MLK for asthma control days (64.2% vs 52.5%; p = 0.004) and for all other control outcomes [51].

Another independent study sponsored by the National Heart, Lung, and Blood Institute tried to identify phenotypic characteristics retaining predictive value for the difference in treatment responses between twice daily IFP and once-daily MLK. Data from the Pediatric Asthma Controller Trial (PACT) were assessed with multivariate analysis. The authors concluded that physicians treating children with parental history of asthma, elevated eNO, low methacholine PC20, or history of ICS use can expect the best long-term outcomes with ICS therapy, as compared to treatment with LTRAs [52].

Another study of Szefler et al. (sponsored by AstraZeneca) compared the long-term efficacy and safety of budesonide inhalation suspension and MLK in children 2 to 8 years old with mild asthma or recurrent wheezing randomized to once-daily budesonide inhalation suspension 0.5 mg or once-daily oral MLK 4 or 5 mg for 52 weeks. No significant differences between-group were observed for time to first additional asthma medication at 52 weeks; however, time to first additional asthma medication was longer at 12 weeks and exacerbation rates were lower over a period of 52 weeks for budesonide versus MLK. Time to first severe exacerbation (requiring oral corticosteroids) was similar in both groups, but the percentage of subjects requiring oral corticosteroids over a period of 52 weeks was lower with budesonide (25.5% vs 32.0%). Peak flow and caregiver and Physician Global Assessments favoured budesonide [53].

## Discussion

The PRACTALL consensus report [21] recommends ICSs as the first-line treatment for persistent asthma, for their capacity to improve symptoms, lung function, airway hyperresponsiveness and to reduce frequency and severity of asthma exacerbations. Atopy and poor lung function predict a favourable response to ICSs [39]. ICSs have potent anti-inflammatory effects in asthmatic airways and, in particular, they reduce eosinophilic airway inflammation.

If control is inadequate on a low dose after 1–2 months, reasons for poor control should be identified and , if indicated, an increased ICS dose or additional therapy with LTRAs or LABA should be considered. It has been known for many years that the effect of ICSs in older children begins to disappear as soon as treatment is discontinued [57]. New evidences do not support a disease-modifying role after cessation of treatment with ICSs in preschool children [21,58-60].

About LTRAs, this consensus [21] establish that they are an alternative first-line treatment for persistent asthma, because evidences support the use of oral MLK as an initial controller therapy for mild asthma in children [6], as it provides bronchoprotection [61] and it reduces airway inflammation as measured by eNO levels in some preschool children with allergic asthma [62,63]. Younger age (< 10 years) and high levels of urinary leukotrienes predict a favourable response to LTRA [39]. MLK is a useful therapy also as add-on therapy to ICS, as the mechanisms of action of the two drugs are different and complementary [37]. Benefit has been shown in children as young as 6 months of age [63,64]. MLK can be a good treatment for viral-induced wheeze, to reduce the frequency of exacerbations in young children aged 2–5 years [65,66] and there is some evidence that they may be beneficial in the 0–2 age group [21,63].

First line or add-on treatment of oral MLK in preschool children with mild to moderate asthma and elevated eNO, decreases eNO levels and improves airway responsiveness, lung function and symptom scores [67].

However ICSs are the current mainstay of treatment in patients with persistent asthma. Several studies, comparing the efficacy of ICSs and LTRAs in patients with persistent asthma, have demonstrated overall greater efficacy of low-dose ICSs for most outcomes of asthma control [16,39,49-53,68-70]. ICSs improve lung function and airway responsiveness to inhaled methacholine, which is the most widely used method of measuring airway hyperresponsiveness in patients with asthma [39,50-52,68,71,72].

However, there is variability in the individual responses of asthmatic patients to these classes of antiasthmatic drugs: higher bronchodilator use, increased bronchodilator responsiveness, higher eNO and eosinophil cationic protein levels, greater airway hyperresponsiveness and pulmonary function values are associated with a greater response to ICS treatment in children. Indeed some authors identified characteristics of patients that should guide the clinician in the choice of asthma control medication: children who have reduced pulmonary function or high levels of markers indicating allergic inflammation should receive ICS therapy [39,68].

As about a quarter of the patients may benefit more from MLK than IFP, therapy with this LTRA could be useful in children with mild-non atopic asthma and/or mainly exercise-induces symptoms [55] and in children with decelerated or poor growth.

Inhibition of linear growth (height) in children has been observed with the administration of ICSs, especially with dosage > 200 mcg/daily [6,57,73-76].

MLK is the first antileukotriene agent available for children 2 to 5 years of age with persistent asthma; it has shown efficacy as a preventive treatment for asthma in a number of clinical trials in children aged 2 to 14 years. The onset of action of MLK is rapid as significant improvements in daytime symptoms are recorded within 1 day. Clinical trials data suggest that MLK effectively counteracts exercise-induced bronchoconstriction and provides protection against bronchoconstriction induced by hyperventilation with cold dry air in 3 to 5 year-old children [77].

Asthma control and pulmonary and inflammatory response improve consistently and significantly also with MLK. Various studies compared its efficacy versus ICSs, without finding clinical and functional differences between drugs [40-48].

Important issues to consider in the treatment of preschool children with asthma are the ease of drug administration and the long-term tolerability of therapy, because treatment is typically chronic. Inhalants are the most commonly prescribed controller therapies; however, very young patients may have difficulty using inhaled corticosteroids and dose delivery can be variable [78-80]. Moreover, reduced compliance with inhalants for asthma compared to orally administered therapy has been reported [6,81].

To optimize beneficial effects from therapy and asthma control, it is very important to adopt a “patient-centered” treatment, i.e. a close relationship between physician and patient, so that the latter can discuss and understand the disease and express preferences for the diagnostic and therapeutic item. Such a relationship implies also that the outcomes are more patient-focused and representative of the patient’s feelings, perceptions and wishes, so enhancing patient adherence to treatment and therapeutic effectiveness [82].

One potential advantage of MLK is the ease of administering a once-daily chewable tablet. Moreover, no tachyphylaxis or change in the safety profile is evident after up to 140 weeks of MLK therapy in adults and 80 weeks of MLK therapy in pediatric patients aged 6 to 14 years [6,83,84].

## Conclusions

Although it is important to recognize that the use of ICSs is currently the recommended first-line treatment for children with asthma, many studies discussed in this article suggest that MLK can have consistent benefit in controlling asthmatic symptoms and may be an alternative, safe, orally administered, non steroidal agent for treating mild persistent asthma, especially in younger children unable to use ICS, not compliant, or victims of adverse effects, or suffering from poor growth. On the contrary, low pulmonary function and/or high allergic inflammatory markers require the corticosteroid use.

## Competing interests

The authors declare that they have no competing interests.
